# QardioArm Upper Arm Blood Pressure Monitor Against Omron M3 Upper Arm Blood Pressure Monitor in Patients With Chronic Kidney Disease: A Validation Study According to the European Society of Hypertension International Protocol Revision 2010

**DOI:** 10.2196/14686

**Published:** 2019-12-02

**Authors:** Victoria Mazoteras-Pardo, Ricardo Becerro-De-Bengoa-Vallejo, Marta Elena Losa-Iglesias, Daniel López-López, David Rodríguez-Sanz, Israel Casado-Hernández, Cesar Calvo-Lobo, Patricia Palomo-López

**Affiliations:** 1 School of Nursing, Physiotherapy, and Podiatry Universidad Complutense de Madrid Madrid Spain; 2 Faculty of Health Sciences Universidad Rey Juan Carlos Madrid Spain; 3 Research, Health, and Podiatry Group, Department of Health Sciences Faculty of Nursing and Podiatry Universidade da Coruña Ferrol Spain; 4 University Center of Plasencia Universidad de Extremadura Plasencia Spain

**Keywords:** blood pressure, hypertension, kidney disease, mobile apps, software validation

## Abstract

**Background:**

Hypertension is considered as a main risk factor for chronic kidney disease development and progression. Thus, the control and evaluation of this disease with new software and devices are especially important in patients who suffer from chronic kidney disease.

**Objective:**

This study aimed to validate the QardioArm mobile device, which is used for blood pressure (BP) self-measurement in patients who suffer from chronic kidney disease, by following the European Society of Hypertension International Protocol 2 (ESH-IP2) guidelines.

**Methods:**

A validation study was carried out by following the ESH-IP2 guidelines. A sample of 33 patients with chronic kidney disease self-measured their BP by using the QardioArm and Omron M3 Intellisense devices. Heart rate (HR), diastolic BP, and systolic BP were measured.

**Results:**

The QardioArm fulfilled the ESH-IP2 validation criteria in patients who suffered from chronic kidney disease.

**Conclusions:**

Thus, this study is considered as the first validation using a wireless upper arm oscillometric device connected to an app to measure BP and HR meeting the ESH-IP2 requirements in patients who suffer from chronic kidney disease. New validation studies following the ESH-IP2 guidelines should be carried out using different BP devices in patients with specific diseases.

## Introduction

### Background

Increased afferent sympathetic activation may be an early event in patients who suffer from chronic kidney disease [1]. Various types of kidney damage may lead to a heightened sympathetic drive by central integrative pathways to the hypothalamus [2,3]. The ensuing efferent response may lead to an increase in renin activity, retention of sodium retention, and, eventually, vasoconstriction, which may contribute to hypertension development and propagation [4]. Hypertension may be considered as a main risk factor for chronic kidney disease development and progression. Thus, the control and evaluation of this disease with new software and devices are especially important in patients who suffer from chronic kidney disease. Patients who suffer from chronic kidney disease must strictly control their hypertension. Nevertheless, most of these patients failed to control their blood pressure (BP), showing a lower control rate compared with the general population [4-6].

Active involvement is required in patients with hypertension to get a successful management of this disease. Encouragement of home BP monitoring is considered as one of the main measures that increased patient compliance with their treatment, showing a great potential to improve hypertension control rates [7-9]. To get efficient home BP monitoring, an accurate BP measurement technique needs to be used by a validated device [7]. Standard validation protocols are considered as objective guidelines, which allow health care professionals to recommend a device to their patients [10-13].

The Association for the Advancement of Medical Instrumentation published a protocol to validate electronic and aneroid sphygmomanometers in 1987. In 1990, the protocol of the British Hypertension Society appeared as a new guideline. Afterward, both protocols were revised in 1993 [10,11]. On the basis of these experiences, the Working Group on Blood Pressure Monitoring of the European Society of Hypertension published a simplified international protocol to facilitate this assessment process in 2002 to revise, unify, and simplify the previous protocols [12]. In 2010, this last European Society of Hypertension protocol was revised (ie, European Society of Hypertension International Protocol 2 [ESH-IP2]), being more exigent than the previous protocol [13]. These protocols have been validated for the general adult population; nevertheless, their validation needs to be carried out in special populations, such as patients with chronic kidney disease [14].

### Objectives

The hypothesis of this study was that QardioArm (Atten Electronics Co) would be valid for self-measurement of BP and heart rate (HR) in renal patients according to the ESH-IP2 guidelines. Hence, the purpose of this study was to validate the QardioArm for BP self-measurement in patients who suffer from chronic kidney disease, following the ESH-IP2 guidelines.

## Methods

### Study Design

This study was a descriptive investigation study to validate the QardioArm device for the measurement of BP and HR in patients with chronic kidney disease according to the ESH-IP2 guidelines [13]. It was performed between January 2019 and May 2019.

### Ethical Information

The Institutional Research and Ethical Committee at the University of Extremadura (Badajoz, Spain), with the code 151/2019, approved this study. This study adhered to the Declaration of Helsinki [15]. Participants were fully informed about the study protocol. All participants signed their written informed consent to participate in this study.

### Devices

#### Omron M3 Intellisense

The Omron M3 Intellisense (Omron Healthcare) was considered as the gold standard in this study. This device was validated in the general population [16] and in patients with chronic kidney disease [14] following the ESH-IP2 guidelines. This device has been validated in comparison with a mercury sphygmomanometer with a mean of −1.3 mm Hg (SD 4.3) for systolic pressure and a mean of 2.1 mm Hg (SD 4.1) for diastolic pressure in patients with chronic kidney disease [14]. In addition, at the beginning of this study, the Omron M3 was evaluated in comparison with a certified pressure device (the Omron M2) [16] in 3 BP measurements to ensure the correct functioning of the gold standard. The used Omron M3 Intellisense monitor was purchased in a local market. The Omron M3 Intellisense is an automated and oscillometric upper arm device for home BP monitoring. This device comprised a standard arm cuff, circumference ranging from 22 to 32 cm, and a large cuff, circumference ranging from 32 to 42 cm. This device used *IntelliSense* technology to acquire a comfortable controlled inflation without pressure presetting or reinflation.

#### QardioArm

The QardioArm was selected as the test device in this study. QardioArm is a fully automatic, noninvasive, wireless BP monitor. QardioArm comprises a BP measurement system intended to assess the diastolic BP and systolic BP and HR in the adult population [17].

This device used an inflatable cuff that was wrapped around the upper arm. Cuff circumference ranged from 22 to 37 cm.

A specific free Qardio app was downloaded from the Apple App Store or Google Play Store. A device with Bluetooth 4.0, iOS 7.0 (or later), and Android 4.4 *KitKat* (or later) was required, being compatible with iPod, iPhone, Apple Watch, iPad, and Android phones and tablets.

Furthermore, the QardioArm provided an automatic screen, including graphics, to facilitate visual data interpretation. This app is configured by issue reminders and warnings, and the measurements and progress are real time shared with other users.

### Patients and Recruitment

All patients were recruited from the *Fresenius Medical Care* dialysis clinics in Plasencia (Extremadura) and signed the written informed consent.

Following the ESH-IP2 guidelines [13], 33 patients who fulfilled the selection criteria were included in this study. Inclusion criteria were women and men, aged at least 25 years, who underwent hemodialysis treatment. Of the total participants, this study included at least 10 men and 10 women, according to the requirements of the guidelines. Exclusion criteria were patients with a sustained arrhythmia or circulatory problems, which are considered as contraindicated conditions for the use of the cuff, as well as pregnant women.

### Study Protocol

A total of 2 nurses with experience in BP measurement carried out all assessments. The measurement room provided an adequate temperature without any factor that could have influenced the measurements, such as noise and distractions [12,13].

Each participant self-reported birth date, sex, height, weight, and body mass index (using the Quetelet index in kg/m^2^), and the arm circumference was measured to ensure the adequate cuff size.

Furthermore, participants were placed in sitting position in the measurement room, and BP measurements were assessed after a rest period (from 10 to 15 min). BP coinciding with the HR was measured on the right arm in 30 patients with chronic kidney disease, whereas BP was assessed on the left arm in 3 patients because of the presence of an arteriovenous fistula on the right arm (n=2) and right hemiplegia (n=1). A total of 9 consecutive measurements were carried out following the ESH-IP2 guidelines [12,13], alternating the 2 described devices (the Omron M3 Intellisense and the QardioArm). All measurements were recorded according to the following protocol:

- BP A—entry BP using the standard device
- BP B—device detection BP using the test instrument
- BP 1—using standard device
- BP 2—using the test instrument
- BP 3—using standard device
- BP 4—using the test instrument
- BP 5—using the standard device
- BP 6—using the test instrument
- BP 7—using the standard device

At the same time of measurement, the patients remained quiet, calm, sitting and without moving, placing the back straight, maintaining the feet over the floor in parallel position, without crossing their legs, and resting the arm over a flat surface, with the hand palm upward and the elbow in a slightly flexed position to place their fist at the height of the heart. The interval time between BP measurements varied from 30 to 60 seconds [13]. All measurements were performed in the same room.

### Data Analysis

Statistical analysis was performed by using IBM SPSS Statistics, version 19 (SPSS Inc). Results were described in mean (SD).

The device accuracy following the ESH-IP2 guidelines was based on a comparison between the measurements of the reference (Omron M3) and test device (QardioArm).

For each patient, the device measurements such as BP 2, BP 4, and BP 6 were first compared with the measurements such as BP 1, BP 3, and BP 5, respectively, and also with the measurements such as BP 3, BP 5, and BP 7, respectively. Comparisons that were more favorable to the device were used.

Indeed, differences were classified separately for both diastolic BP and systolic BP, depending on whether their values were within 5, 10, or 15 mm Hg [13], and for HR, depending on whether their values were within 3, 5, or 8 beats per minute.

Results were analyzed and detailed according to the ESH-IP2 requirements to conclude if the used device passed or failed to pass the explained validation protocol. Parts 1 and 2 of the validation protocol concern the differences in number of the requested ranges for each individual measurement (99 measurements) and each individual patient (33 patients), respectively [13].

Furthermore, Bland and Altman graphs were used to illustrate the relationship between systolic BP differences (systolic BP and device-reference) and mean systolic BP (device and reference), diastolic BP differences (diastolic BP and device-reference) and mean diastolic BP (device and reference), or HR differences (HR and device-reference) and average HR (device and reference).

## Results

### Patients With Chronic Kidney Disease

A sample of 35 patients with chronic kidney disease were recruited to assess 33 participants who met the ESH-IP2 inclusion criteria, and 2 of them were excluded because of device failure (n=1) and arrhythmias (n=1).

The remaining sample (n=33) was screened. There were 15 females and 18 males. The characteristics of the patients, such as age, height, weight, body mass index, and arm circumference, are presented in Table 1.

**Table 1:** Sociodemographic characteristics of the patients.

Variables	Total group (N=33)		Male (n=18)		Female (n=15)	
	Mean (SD)	Range (minimum to maximum)	Mean (SD)	Range (minimum to maximum)	Mean (SD)	Range (minimum to maximum)
Age (years)	71.03 (11.24)	45.0-91.0	70.11 (11.11)	45.0-90.0	72.13 (11.69)	48.0-91.0
Weight (kg)	70.70 (15.68)	46.5-101.0	70.33 (13.66)	46.5-100.0	71.15 (18.31)	46.80-101.0
Height (cm)	162.30 (9.52)	141.0-180.0	166.61 (5.63)	155.0-180.0	157.13 (10.77)	141.0-174.0
Body mass index (kg/m^2^)	27.02 (6.70)	18.07-43.72	25.26 (4.27)	18.07-33.48	29.14 (8.48)	18.96-43.72
Arm circumference (mm)	267.27 (31.18)	215.0-350.0	262.50 (24.15)	220.0-310.0	273.0 (38.06)	215.0-350.0

### Blood Pressure Outcome Measurements

Validation findings for the QardioArm BP device following the 2010 ESH-IP2 are presented in Table 2 (Part 1), Table 3 (Part 2) and Textbox 1 (Part 3).

The measurement numbers differing from the standard device (Omron M3) of 5, 10, and 15 mm Hg or less were presented in Tables 2 and 3 and Textbox 1, for diastolic BP and systolic BP, following the ESH-IP2 [13].

Mean differences between the test device and standard device were 2.43 mm Hg (SD 4.15) for diastolic BP and 4.03 mm Hg (SD 4.42) for systolic BP.

From these analyses, of 99 measurements, 81 differences for systolic BP and 85 differences for diastolic BP showed an absolute difference within 5 mm Hg (compared with at least 65 differences for diastolic BP and 73 differences for systolic BP according to the ESH-IP2 criteria). Furthermore, 92 comparisons for systolic BP and 95 comparisons for diastolic BP showed an absolute difference within 10 mm Hg (compared with at least 81 differences for diastolic BP and 87 differences for systolic BP according to the ESH-IP2 criteria).

In addition, of 99 differences, 96 for systolic BP and 95 for diastolic BP exhibited an absolute difference within 15 mm Hg (compared with at least 93 for diastolic BP and 96 for systolic BP according to the ESH-IP2 criteria). Indeed, the validation of part 1 of the device was successfully completed.

According to part 2 of the 2010 ESH-IP2 criteria, of 33 patients, 29 patients showed a minimum of 2 of 3 comparisons within a 5 mm Hg difference for systolic BP, and 30 patients showed a minimum of 2 of 3 comparisons within a 5 mm Hg difference for diastolic BP (compared with at least 24 patients for systolic BP and diastolic BP according to the ESH-IP2 criteria). Nevertheless, 2 patients showed their 3 differences outside 5 mm Hg for systolic BP, and no patients showed their 3 differences outside 5 mm Hg for diastolic BP (compared with a maximum of 3 patients for diastolic BP and systolic BP according to the ESH-IP2 criteria). Owing to these 2 described conditions, validation of part 2 of the device was successfully completed.

Therefore, part 3 of the QardioArm device validation was completed, as both parts 1 and 2 were validated for diastolic BP and systolic BP.

**Table 2:** Validation results of the Part 1 of the QardioArm blood pressure device according to the European Society of Hypertension International Protocol 2010.

Validation results of QardioArm—Part 1^a^			≤5 mm Hg		≤10 mm Hg		≤15 mm Hg		Grade 1		Mean (SD), mm Hg
**Pass requirements^b^**											
	Two of	73		87		96		—^c^		—	
	All of	65		81		93		—		—	
**Achieved^d^**											
	Systolic blood pressure	81		92		96		Pass		4.03 (4.42)	
	Diastolic blood pressure	85		95		95		Pass		2.43 (4.15)	

^a^Accuracy is determined by the number differences in these ranges for both individual measurements (part 1) and individual subjects (part 2). To pass, a device must achieve all the minimum pass requirements shown.

^b^Pass requirements: as required by the IP.

^c^Not applicable.

^d^Achieved: as recorded by the device.

**Table 3:** Validation results of the Part 2 of the QardioArm blood pressure device according to the European Society of Hypertension International Protocol 2010.

Validation results of QardioArm—Part 2^a^		2/3≤5 mm Hg	0/3≤5 mm Hg	Grade 2	Grade 3
Pass requirements^b^		≥24	≤3	—^c^	—
**Achieved** ^d^					
	Systolic blood pressure	29	2	Pass	Pass
	Diastolic blood pressure	30	0	Pass	Pass

^a^Accuracy is determined by the number differences in these ranges for both individual measurements (part 1) and individual subjects (part 2). To pass, a device must achieve all the minimum pass requirements shown.

^b^Pass requirements: as required by the IP.

^c^Not applicable.

^d^Achieved: as recorded by the device.

**Validation results of the Part 3 of the QardioArm blood pressure device according to the European Society of Hypertension International Protocol 2010.**

Validation results of QardioArm—Part 3

- Result: Pass

### Heart Rate Outcome Measurements

Validation findings for the QardioArm HR device following the 2010 ESH-IP2 are presented in Table 5 (Part 1), Table 6 (Part 2) and Textbox 2 (Part 3).

Measurement numbers differing from the standard device Omron M3 of 3, 5, and 8 beats per minute or less are detailed in Tables 5 and 6 and Textbox 2 for HR. Mean differences between the test device and standard device were 1.93 beats per minute (SD 3.04).

From these analyses, of 99 differences, 85 showed an absolute difference within 3 beats per minute, 94 differences showed an absolute difference within 5 beats per minute, and 95 differences showed an absolute difference within 8 beats per minute. Thus, part 1 device validation was successfully completed for the HR.

According to the part 2 of the 2010 ESH-IP2, of 33 participants, 29 showed a minimum of 2 of 3 comparisons within 3 beats per minute difference for HR. Nevertheless, 1 participant showed 3 differences outside 3 beats per minute. As these 2 detailed conditions were validated, the part 2 device validation was successfully completed.

Therefore, part 3 of the QardioArm device validation was completed, as both parts 1 and 2 were validated for HR.

Indeed, the QardioArm device met the validation criteria of the ESH-IP2 for the diastolic BP, systolic BP, and HR for patients who suffered from chronic kidney disease.

**Figure 1:**
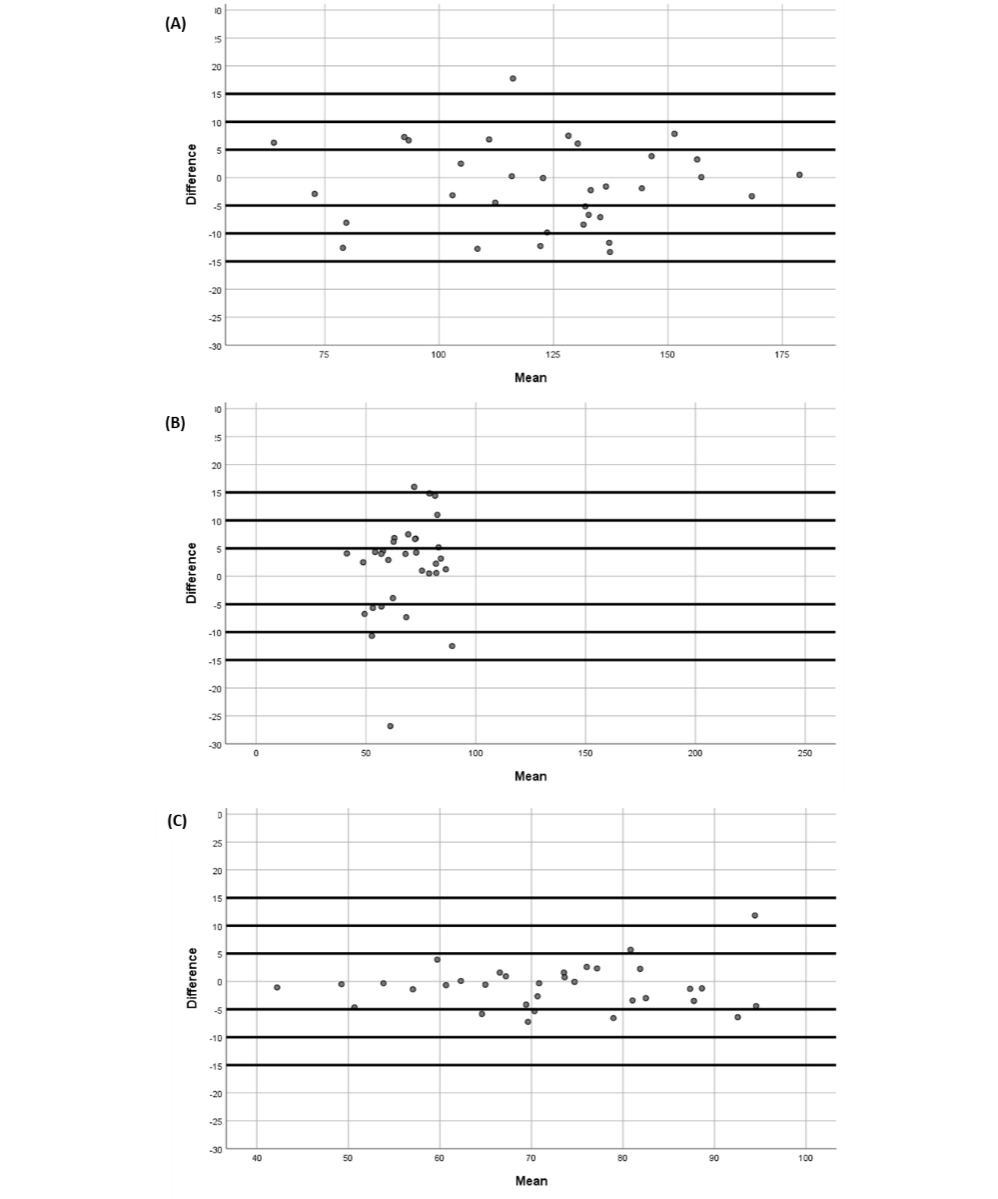
Plots of (A) systolic blood pressure, (B) diastolic blood pressure, and (c) heart rate differences between the QardioArm and Omron M3. Difference: systolic blood pressure (A), diastolic blood pressure (B), or heart rate (C) differences between the QardioArm and Omron M3. Mean: mean systolic (A), diastolic (B), or heart rate (C) average values of the QardioArm and Omron M3.

The prior findings coincided with the Bland and Altman graphs that visually showed the differences between QardioArm device measurements and Omron M3 measurements for systolic BP (Figure 1), diastolic BP (Figure 1) and HR (Figure 1).

**Table 5:** Validation results of the Part 1 for the QardioArm heart rate device according to the European Society of Hypertension International Protocol 2010.

Validation results QardioArm—Part 1^a^		≤3 bpm	≤5 bpm	≤8 bpm	Grade 1	Mean (SD), bpm
**Pass requirements^b^**						
	Two of	73	87	96	—^c^	—
	All of	65	81	93	—	—
**Achieved^d^**						
	Heart rate	85	94	95	Pass	1.93 (3.04)

^a^Accuracy is determined by the number differences in these ranges for both individual measurements (part 1) and individual subjects (part 2). To pass, a device must achieve all the minimum pass requirements shown.

^b^Pass requirements: as required by the IP.

^c^Not applicable.

^d^Achieved: as recorded by the device.

**Table 6:** Validation results of the Part 2 for the QardioArm heart rate device according to the European Society of Hypertension International Protocol 2010.

Validation results QardioArm—Part 2^a^		2/3≤3 bpm	0/3≤3 bpm	Grade 2	Grade 3
Pass requirements^b^		≥24	≤3	—^c^	—
**Achieved^d^**					
	Heart rate	29	1	Pass	Pass

^a^Accuracy is determined by the number differences in these ranges for both individual measurements (part 1) and individual subjects (part 2). To pass, a device must achieve all the minimum pass requirements shown.

^b^Pass requirements: as required by the IP.

^c^Not applicable.

^d^Achieved: as recorded by the device.

**Validation results of the Part 3 for the QardioArm heart rate device according to the European Society of Hypertension International Protocol 2010.**

Validation results QardioArm—Part 3

- Result: Pass

## Discussion

### Principal Findings

Globally, the use of home BP monitoring is increasing in several countries, being a useful complement to clinic measurements with significant acceptance by patients with hypertension with several advantages [7,9,18,19]. Patients who suffer from chronic kidney disease may use a validated sphygmomanometer at home because it seems to be especially cost-effective [6,7,18,20-22].

The main disadvantage of automated home sphygmomanometers is their inaccuracy, although their accuracy is progressively improving [13]. This inaccuracy is more frequent in populations with specific diseases, which may require additional validation tests [7,23]. Indeed, the European Society of Hypertension Practice Guidelines 2010 for home BP monitoring recommended specific validation tests for patients with end-stage chronic kidney disease [9]. Arterial stiffness may influence the correspondence between readings measured by using mercury and oscillometric devices [8,24].

Nevertheless, there is a lack of research studies, which validate devices in patients with chronic kidney disease [14,24-27]. Further validation studies are necessary for patients with chronic kidney disease. Indeed, further studies should specifically investigate the validation of QardioArm in patients with chronic kidney disease with arterial stiffness as a future line of research.

This research is considered as the first study investigating the validation of a wireless upper arm oscillometric device connected to an app to measure HR and BP in chronic kidney disease patients. This validation has been carried out following the ESH-IP2 guidelines, although a validated noninvasive oscillometric upper arm device was used as a reference instead of a mercury sphygmomanometer.

QardioArm has been previously validated for the general population in the first place by our team [28] and later by other authors [29]. In addition, our team validated QardioArm in obese patients [30].

According to the results of our prior works, the number of differences included in each category according to the ESH-IP2 (5, 10, and 15 mm Hg) for systolic BP and diastolic BP was similar in the 3 validations [28-30], as parts 1 and 2 of the protocol were passed. QardioArm in the general population achieved better results in both phases of the protocol for systolic and diastolic BP, especially in part 1 (higher differences in the 3 categories) [28]. Within phase 1, the differences obtained in the systolic BP of renal patients in this study were very similar to those of the general population [28], whereas the differences obtained in the diastolic BP were more similar to the obese population [30]. Phase 2 of this study was almost identical to the 3 previous validations [28-30], with minor differences (1 or 2 individuals).

Following the ESH-IP2 guidelines, the findings of this study showed that the QardioArm device successfully passed the validation requirements for patients with chronic kidney disease [13]. Nevertheless, our findings may not be extrapolated to other specific populations with specific diseases such as elderly or diabetic patients as well as pregnant women, as these conditions have not been addressed. In addition, it should be considered that patients with advanced chronic kidney disease could present a specific chronic kidney disease type and future studies should be carried out to develop new app validations according to the specific recommendations in each kind of chronic kidney disease patients Nevertheless, arterial stiffness measurements of patients with chronic kidney disease involved in this study could be useful, although the standard validation protocols did not require these measures. Finally, consecutive sampling bias should be considered in this study, and a simple randomization sampling process could be more adequate for future studies.

### Conclusions

The findings of this study are relevant because it is considered as the first validation to show that a device connected to an app to measure BP and HR met the requirements of the 2010 ESH-IP2 in the patients who suffer from chronic kidney disease.

Besides, the ESH-IP2 guidelines should stress on validating the BP devices in other specific populations by publishing explicit criteria for such a validation in these populations.

Finally, it is highly recommended to determine the accuracy of this device in other populations with specific diseases such as pregnant women, elderly people, or arrhythmic patients.

## Abbreviations

BP: blood pressure
ESH-IP2: European Society of Hypertension International Protocol 2
HR: heart rate
